# Prognostic Indicator for Pleural Mesothelioma

**DOI:** 10.1016/j.atssr.2024.05.011

**Published:** 2024-06-06

**Authors:** Masaya Yotsukura, Yukihiro Yoshida, Kazuo Nakagawa, Shun-ichi Watanabe

**Affiliations:** 1 Department of Thoracic Surgery, National Cancer Center Hospital, Tokyo, Japan

## Abstract

**Background:**

In malignant pleural mesothelioma), it is difficult to evaluate the degree of tumor progression using imaging findings. It is essential to develop an objective index that is independent of imaging findings and useful for assessing the degree of tumor progression and indications for surgery.

**Methods:**

We retrospectively evaluated the data of 79 patients with malignant pleural mesothelioma who underwent extrapleural pneumonectomy or pleurectomy/decortication at our institution between 1999 and 2022. The postoperative prognosis was evaluated based on clinical factors.

**Results:**

Of the 79 patients, extrapleural pneumonectomy was performed in 41 (51.9%), and pleurectomy/decortication was performed in 38 (48.1%). Univariate analyses identified that percent predicted forced vital capacity (FVC) < 80% (*P* < .01), rind-like growth pattern on computed tomography (*P* < .01), Glasgow Prognostic Score ≥ 1 (*P* < .01), and pathologic stage ≥ II (*P* < .01) were poor prognostic factors for overall survival. In the multivariate analysis, percent predicted FVC <80% (hazard ratio, 2.76; 95% CI, 1.23-6.18, *P* = .01) was found to be the only poor prognostic factor for overall survival after surgery. Pathologic stage was a less significant prognostic factor (hazard ratio, 1.83; 95% CI, 0.95-3.53, *P* = .07). Two-year overall survival in patients with percent predicted FVC ≥80% and <80% was 76.6% ± 6.6% and 16.6% ± 7.5%, respectively.

**Conclusions:**

FVC is a strong predictor of postoperative survival in patients with malignant pleural mesothelioma independent of imaging findings. FVC is useful for assessing tumor invasion and would help determining surgical indication.

## Visual Abstract

**Figure:**
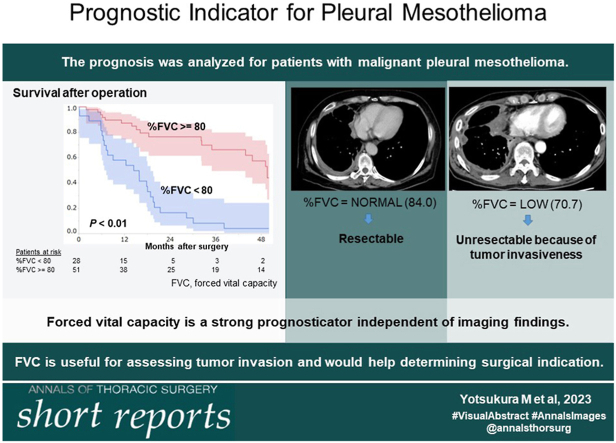

**In Short**

▪ Radiologic evaluation for tumor invasion of mesothelioma is difficult.
▪ Forced vital capacity is a strong prognosticator independent of imaging.
▪ Low forced vital capacity reflects tumor invasion and even resectability.

Malignant pleural mesothelioma (MPM) is a tumor for which prognostic stratification by TNM classification is difficult.^1^^,^^2^ It is not easy to evaluate the invasion into adjacent structures using imaging modalities because the anatomical extension of MPM is not necessarily in a form that can be recognized as a mass or nodule.^3^, ^4^, ^5^ Tumor thickness is a concern for interobserver discrepancy due to uneven thickness of the tumor.^2^^,^^6^ Difficulty in staging might be one of the causes of the difficulty in determining treatment strategies for MPM. Therefore, it is imperative to develop a simple and easily accessible prognostic factor that can be beneficial in determining treatment strategies. This study aimed to identify an appropriate objective clinical factor that can be useful in the clinical setting of MPM.

## Material and Methods

### Patient Collection and Histologic Evaluation

We retrospectively collected data from 79 patients with MPM who underwent maximal cytoreductive surgery, either by extrapleural pneumonectomy (EPP) or pleurectomy/decortication (P/D), between 1999 and 2022 at the National Cancer Center Hospital, Tokyo, Japan. Patients who underwent partial pleural resection were excluded. All clinical information was extracted from patients’ medical records. The patients’ age at operation, sex, tumor location, exposure to asbestos, pulmonary function, computed tomography findings, perioperative chemotherapy and radiotherapy, and stage classification according to the TNM criteria from the 8th edition of the Union for International Cancer Control classification were collected (shown in Table 1, Table 2).

**Table 1:** Characteristics of the Included Patients

Factor	Value
Number of patients, N	79
Sex	
Male	64 (81.0)
Female	15 (19.0)
Age, y, median (interquartile range)	61 (55-68)
Side	
Left	31 (39.2)
Right	48 (60.8)
Exposure to asbestos	
Present	36 (45.6)
Absent	–54.4
Percent predicted FVC	
< 80	28 (35.4)
≥ 80	51 (64.6)
EORTC prognostic score	
Low risk	67 (84.8)
High risk	12 (15.2)
Glasgow Prognostic Score	
1 or more	26 (32.9)
0	53 (67.1)
CT findings	
Rindlike	20 (25.3)
Other	59 (74.7)
Operative procedure	
EPP	41 (51.9)
P/D	38 (48.1)
Neoadjuvant chemotherapy	
Present	16 (20.3)
Absent	63 (79.7)
Adjuvant chemotherapy	
Present	12 (15.2)
Absent	67 (87.8)
Postoperative radiotherapy	
Present	25 (31.6)
Absent	54 (68.4)
Clinical stage	
I	72 (91.1)
II	2 (2.5)
III	5 (6.3)
Pathologic stage	
I	48 (60.8)
II	8 (10.1)
III	23 (29.1)
Histology	
Epithelioid	49 (62.0)
Biphasic or sarcomatoid	30 (38.0)

Values are presented as n (%) unless otherwise noted.

CT, computed tomography; EORTC, European Organisation for Research and Treatment of Cancer; EPP, extrapleural pneumonectomy; FVC, forced vital capacity; P/D, pleurectomy/decortication.

**Table 2:** Univariate and Multivariate Analyses of Factors Affecting Overall Survival After Surgery

Characteristics	Univariate Analysis		Multivariate Analysis	
	2-year Overall Survival Probability (%) (95% CI)	*P* Value	Hazard Ratio (95% CI)	*P* Value
Age				
>60 y	39.8 (22.6-57.0)	.11		
≤60 y	66.8 (50.6-83.1)			
Sex				
Male	51.7 (38.1-65.3)	.20		
Female	61.0 (33.1-88.8)			
Pleural effusion				
Present	47.9 (33.8-61.9)	.06		
Absent	72.2 (49.0-95.5)			
Side				
Right	52.7 (36.7-68.6)	.96		
Left	55.0 (35.8-74.1)			
Performance status				
≥1	26.9 (1.4-52.5)	.04		
0	60.0 (46.7-73.2)			
Exposure to asbestos				
Present	66.7 (50.0-83.8)	.46		
Absent	42.5 (25.8-59.1)			
Percent predicted FVC				
<80	16.6 (1.8-31.3)	<.01	2.76 (1.23-6.18)	.01
≥80	76.6 (63.7-89.5)		1	
Pleural thickness (sum of the upper, middle, and lower levels)				
>25 mm	43.1 (24.5-61.8)	.36		
≤ 25 mm	58.8 (42.3-75.2)			
Rindlike growth				
Present	20.4 (0.2-40.5)	<.01	1.43 (56.4-3.63)	.45
Absent	62.5 (48.5-76.5)		1	
Glasgow Prognostic Score				
≥1	26.4 (6.8-46.0)	<.01	1.73 (0.75-4.00)	.20
0	65.7 (51.8-79.6)		1	
EORTC prognostic score				
High risk	21.4 (0-47.5)	.11		
Low risk	59.3 (46.3-72.4)			
Histology				
Biphasic or sarcomatoid	34.8 (16.7-52.8)	.04	1.06 (0.52-2.15)	.87
Epithelioid	66.9 (51.9-82.0)		1	
Clinical stage				
≥IB	44.8 (28.3-61.2)	.12		
IA	64.5 (47.1-81.8)			
Pathologic stage				
≥II	36.4 (18.1-54.6)	<.01	1.83 (0.95-3.53)	.07
I	66.2 (50.8-81.5)		1	
Operative procedure				
P/D	60.5 (41.6-79.4)	.61		
EPP	48.8 (33.0-64.6)			
Preoperative chemotherapy				
Present	53.6 (22.6-84.5)	.84		
Absent	53.0 (39.6-66.4)			
Adjuvant chemotherapy				
Present	70.0 (41.6-98.4)	.19		
Absent	50.8 (37.4-64.2)			
Postoperative radiotherapy				
Present	56.3 (35.8-76.9)	.77		
Absent	52.2 (36.9-67.5)			

FVC, forced vital capacity; EORTC, European Organisation for Research and Treatment of Cancer; EPP, extrapleural pneumonectomy; P/D, pleurectomy/decortication.

### Scoring

In this study, the European Organization for Research and Treatment of Cancer Prognostic Score (EPS) and Glasgow Prognostic Score (GPS), 2 scoring systems that may be prognostic factors for MPM, were calculated for each case.

The EPS was the sum of 0.55 (if white blood cell count > 8.3 x 10^9^/L), 0.6 (if performance status = 1 or 2), 0.52 (if histologic diagnosis was probable or possible), 0.67 (if histology was sarcomatoid), and 0.6 (if male). The patient had a good prognosis if EPS ≤1.27 and a poor prognosis if EPS >1.27.^7^

GPS was computed based on serum concentrations of C-reactive protein (CRP) and albumin. Patients with an elevated CRP level (>0.3 mg/dL) and a low albumin level (<3.5 mg/dL) were classified as having a score of 2, whereas those with only an abnormal CRP level were given a score of 1. Those with a normal CRP value were given a score of 0, irrespective of the albumin level.^8^^,^^9^

### Patient Follow-up and Statistical Analysis

Tumor recurrence during routine postsurgical follow-up was recorded. Follow-up was conducted until the end of December 2022.

Overall survival (OS) was estimated using the Kaplan-Meier method, and the significance of differences was analyzed using the log-rank test in the univariate setting. Multivariate analysis was performed using Cox proportional hazards model. All tests were 2-sided, and statistical significance was set at *P* value <.05. All statistical analyses were performed using JMP 15 (SAS Institute Inc). Because the surgery for MPM is potentially related to microscopically incomplete resection, we could not use the resectability for the primary outcome, but used the OS. In the univariate and multivariate analyses, clinical stage was separated between stage IA and IB or more because more than 90% of the cases were classified as clinical stage I, whereas pathologic stage was separated between stage I and II or more because approximately 40% of the cases were classified as pathologic stage II or more.

### Ethics Approval

The study protocol was approved by the Medical Research Ethics Committee of the National Cancer Center (IRB approval no. 2022-274), and all experiments were conducted in accordance with the Declaration of Helsinki. The requirement for informed consent was waived by the committee because our study was a retrospective review of the patient records.

## Results

### Patient Demographics and Clinical and Pathologic Characteristics

Clinical and pathologic data of the patients are shown in Table 1. EPP was performed in 41 patients (51.9%), and P/D was performed in 38 patients (48.1%). Upgrades of stage are frequent at both clinical and pathologic stages, suggesting the difficulty in diagnosing the clinical stage of mesothelioma.

In terms of clinical stage, 72 patients (91.1%) were stage I, whereas in terms of pathologic stage 48 (60.8%) were stage I and 331 (39.2%) were stage II or higher. The balance of distribution of the stages was taken into account when separating stages in prognostic analyses.

### Two-year Prognosis After Resection

**Figure 1:**
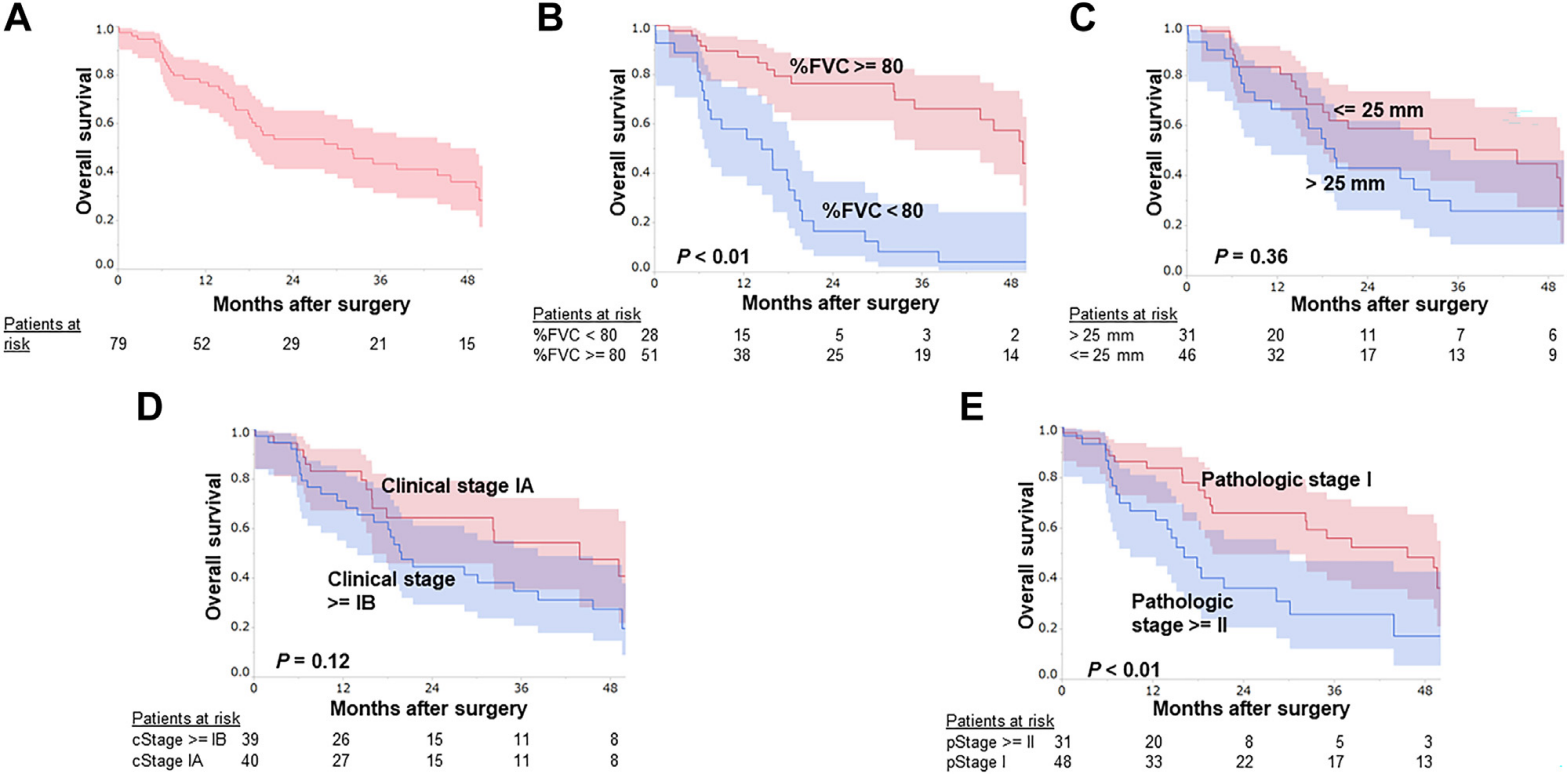
Kaplan-Meier curves of overall survival after surgery for malignant pleural mesothelioma: (A) for all included patients, and stratified by (B) percent predicted forced vital capacity (FVC), (C) pleural thickness, (D) clinical stage, and (E) pathologic stage.

The estimated probability of 2-year OS was 53.6% ± 6.3% in the entire cohort (Figure 1A). For patients with pathologic stage I and ≥ II MPM, the 2-year OS rates were 66.2% ± 7.8% and 36.4% ± 9.3%, respectively (*P* < .01).

### Prognostic Factors After Surgery

Univariate analyses identified that percent predicted FVC of less than 80% (*P* < .01), rind-like growth on computed tomography (*P* < .01), GPS of 1 or more (*P* < .01), and pathologic stage II or higher (*P* < .01) were poor prognostic factors for OS. The Kaplan-Meier curves for each factor are shown in Figures 1b-1e.

Four factors with *P* values less than .05 in the univariate analysis were entered into the multivariate analysis. As a result, a percent predicted FVC of less than 80% (hazard ratio, 2.76; 95% CI, 1.23-6.18; *P* = .01) was found to be the only poor prognostic factor for OS after surgery (Table 2). Two-year OS in patients with a percent predicted FVC of 80% or more was 76.6% ± 6.6%, while it was 16.6% ± 7.5% in patients with a percent predicted FVC of less than 80%.

## Comment

MPM is a tumor that is difficult to assess for local tumor extension, making it difficult to determine the treatment strategy and stratify the prognosis by clinical diagnosis.^2^ In this study, only restrictive lung disorder (percent predicted FVC <80%) was a significant poor postoperative prognostic factor. As far as we know, no previous reports have described restrictive lung disorder as a prognostic factor after surgery for MPM. However, FVC has been used as a measure of treatment response in Response Evaluation Criteria in Solid Tumors (RECiST)^10^; therefore, the results of this study are expected to be reproducible.

**Figure 2:**
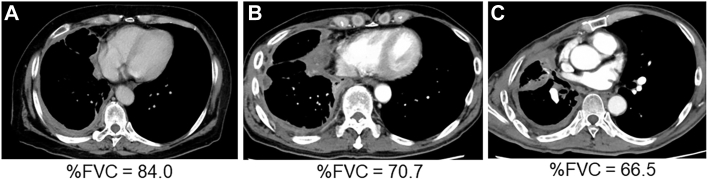
Computed tomography findings of cases with (A) normal (>80%) percent predicted forced vital capacity (FVC) value; and (B, C) decreased (<80%) percent predicted FVC value.

Figure 2A represents a case with a normal (>80%) percent predicted FVC value, and Figure 2B shows a case of a decreased (less than 80%) percent predicted FVC value. Both cases (Figures 2A, 2B) presented with almost the same pleural thickness on computed tomography images; however, their prognoses were supposed to be completely different according to the results of our study. In fact, the tumor in Figure 2B was not resected by EPP or P/D because of severe tumor invasion into the mediastinum and chest wall (Figure 2B was not included as study material in this study). In cases where thoracic contraction is pronounced (Figure 2C), the infiltration is suggested to be strong, even if pleural thickening is not very severe (Figure 2C was not included as study material in this study, because the tumor in Figure 2C was not resected by EPP or P/D). Thus, percent predicted FVC might be able to predict prognosis and surgical resectability that cannot be predicted by pleural thickness.

In this study, GPS and EORTC score were not prognostic factors after surgery. These are indirect prognostic markers of MPM, based on blood tests and clinical information. FVC was considered a more accurate prognostic factor for MPM than indirect markers, because FVC directly reflects thoracic mobility and tumor invasiveness.

Because this study included only operated cases, it should be noted that FVC may not necessarily be a useful prognostic factor in cases with lymph node or distant organ metastasis. The limitations of this study include its single-center retrospective design. Also, there is no uniformity regarding the indications for perioperative multimodality treatment, thus our results do not support that the FVC is be used for judgment of resectability. Finally, one drawback of FVC is that it is affected by other constrictive lung diseases. A larger prospective analysis of the prognostic factors is required for further evaluation.

This study had several strengths. FVC, found in this study to be a clear prognostic factor, is a highly objective and accessible measure. Because the assessment of FVC is readily available worldwide, the results of this study can be applied in clinical settings around the world. It is worth noting that FVC was a stronger prognostic factor than tumor thickness, stage, or inflammation score. When considering surgical indications, if FVC is low, the tumor may be highly invasive and difficult to resect, even if the pleural thickening is mild. When the FVC is in the normal range, the tumor may not be highly invasive, and the indication for surgery may be aggressively considered, even if pleural thickening is severe.

In conclusion, FVC is a clear prognostic factor for MPM after surgery. Unlike tumor thickness and volume, FVC, which can be easily measured objectively and reproducibly, is an indicator with excellent clinical applicability. The use of FVC can be recommended for estimating the prognosis of MPM and determine surgical indication, which cannot be well assessed by TNM classification alone.
